# Exploring environmental sustainability in dentistry among students and educators in the United Arab Emirates: a cross-sectional survey

**DOI:** 10.1186/s12909-024-05488-x

**Published:** 2024-05-02

**Authors:** Mohannad Nassar, Wed Shalan, Uesser Al-Janaby, Hagar Elnagar, Maryam Alawadhi, Sara Jaser, Easter Joury

**Affiliations:** 1 Department of Preventive and Restorative Dentistry, College of Dental Medicine, University of Sharjah, Sharjah, United Arab Emirates; 2 Research Institute for Medical and Health Sciences, University of Sharjah, Sharjah, United Arab Emirates; 3 College of Dental Medicine, University of Sharjah, Sharjah, United Arab Emirates; 4 Centre for Dental Public Health and Primary Care, Institute of Dentistry, Queen Mary University of London, London, UK

**Keywords:** Environmental sustainability, Dentistry, Students, Faculty, Educators, Curriculum

## Abstract

**Objectives:**

Creating environmentally sustainable healthcare culture within the dental field requires embedding the content in the dental curriculum at the undergraduate level. This study aimed to explore the current awareness and drivers among dental students and educators regarding environmentally sustainable dentistry (ESD) in the United Arab Emirates (UAE) and identify barriers and enablers to embrace it.

**Methods:**

A cross-sectional survey using online questionnaires was carried out in six undergraduate dental education institutes within the UAE. Data analysis included descriptive statistics.

**Results:**

In total 153 students and 53 educators participated in the survey. Most students and educators were not aware of any ESD content in their curricula. However, the majority of educators were familiar with the concept of ESD, while students were mostly unfamiliar or slightly familiar. Nonetheless, students largely agreed on its importance and their interest in learning it, as they viewed it relevant to their future practice. Educators agreed that the dental profession has a responsibility to be environmentally friendly and expressed their desire in introducing ESD content into the curricula. Several barriers were reported such as lack of knowledge, curricula space, educational resources, and time. Meanwhile, enablers included providing ESD capacity building, training and resources.

**Conclusions:**

There was no explicit presence of ESD content in the dental curricula in the UAE. Despite the lack of adequate awareness on this topic among educators and more so among students, they both had positive views towards incorporating ESD into dental curricula. Overall, this study highlighted the importance of promoting ESD in dental programs. Clinical significance: ESD is gradually becoming a critical aspect of modern oral healthcare system. It has been mandated in the dental curricula in several regions globally. Embedding ESD in the UAE dental curricula would have several benefits for the environment as well as the future of the dental profession in the region. The clinical significance includes, but not limited to, improved treatment outcomes; patients, students and staff health and well-being; reduced health risks, and cost savings.

## Introduction

Climate change has an impact on human health today and is expected to have a greater effect in the future. Years of research, negotiations, and panel meetings by the United Nations, World Health Organization and Centers for Disease Control and Prevention highlighted the importance of responding to climate change and fighting its global and health effects [1, 2]. Some of which include extreme heat, severe weather, air pollution, food and water-borne illnesses and infectious diseases that can have repercussions on human health like cardiovascular diseases, respiratory problems, and the fast spread of viral infections. The latter can increase the incidence of pandemics and contribute to the difficulty of controlling them such as the recent COVID-19 breakout [3, 4]. As more people are becoming aware of the great threats that climate change imposes on the planet, it is important to comprehend the climate footprint of the healthcare system. Over the decades, the global expansion of the medical sector, combined with the increase in the use of disposable medical products, has contributed to the generation of massive amounts of medical waste [5]. According to the World Economic Forum, the healthcare systems account for over 4% of global carbon dioxide (CO_2_) emission, which is considered more than that of the aviation and shipping sectors combined. Hospitals have the highest energy intensity of all publicly funded buildings and emit 2.5 times more greenhouse gasses than commercial buildings [3]. The global health climate footprint is equivalent to the annual greenhouse gas emissions from 514 coal-fired power plants [6].

The oral healthcare field is also a demanding and extremely resource intensive sector that heavily relies on large supplies of energy, water, and fuel for various clinical activities [7, 8]. In dentistry, there are impacts on the environment including air, water and land pollution and loss of biodiversity along the timeline of the practice, starting from resourcing the materials and products, manufacturing them, the transport of patients to and from the clinic, the energy consumption of the practice, and complex waste management [9]. For instance, Duane et al. (2019) identified the aspects of dental practice that have the highest impact on environmental emissions in England. After categorizing them into core emissions, community emissions, and supply chain emissions, they found that the highest CO_2_ emission in dentistry comes from the community. Travel is the most concerning as it directly contributes to CO_2_ emissions and has an impact on population health and quality-adjusted life years, and to achieve a sustainable route there is a need to identify external areas outside of the dentist’s control that can have an influence on the adoption of sustainable dentistry practices such as policy makers, professional educators, and researchers [10, 11]. Care towards planetary sustainability led to various international policy agreements such as the Paris agreement and the Sustainable Development Goals (SDG) [12]. However, a main hurdle to switch from the established conventional dental practice into a more sustainable one is the low awareness among the profession. The 5th European Dental Students’ Association Congress of Belgrade introduced the term “Green Dentistry” which refers to the concept of environmental sustainability in dentistry (ESD) and recommended several regulations and policies that are compatible with sustainable development strategies [13]. More recently, there is notable growing interest in ESD in the education sector that is evident by conferences, presentations and contributions from student committees and dental organizations worldwide [12–15].

Although ESD started to gain popularity in recent years, related research started worldwide more than a decade ago [16–18]. Fortunately, pioneers in ESD are emerging around the globe, from Europe and Asia; however, if we view the topic from a wider scope, knowledge about ESD is still deficient, and more research is warranted for better understanding of the current situation and broadly disseminating related information. A study from the school of dentistry at the University of Manchester demonstrated that despite students’ familiarity with environmental aspects of sustainability, additional efforts are needed to foster understanding and awareness of sustainability within the dental curriculum [19]. Similarly, a more recent study conducted to explore ESD in dental curricula at two Institutes in the United Kingdom (UK) and the United States of America (USA), highlighted the interest of students and educators in the topic of ESD despite the lack of formal presence of the topic in their dental curricula [20]. According to Martin et al. education plays a paramount role in raising proper awareness and promoting positive behavior and attitude changes for the adoption of policies related to ESD, and the starting point can be at an undergraduate university degree program and further through continuing professional development programs [9]. Gershberg and colleagues underscored the enthusiasm of dental students towards ESD, proposing its potential integration into current curriculum modules covering infection control, practice administration, and dental public health [21]. Research encompassing all dental schools in the UK revealed that most students have contemplated the environmental footprint of oral healthcare provision and acknowledged their obligation, as oral health practitioners, to deliver care in an environmentally sustainable manner. Nonetheless, a prevalent observation was their perceived lack of authority within the institute, with their participation in contributing to this matter being devoid of any significant influence [22]. The World Dental Federation consensus statement and the Association for Dental Education in Europe (ADEE) Special Interest Group not only advocated for the establishment of clear ESD learning outcomes and effective teaching and assessment approaches but also emphasized the critical need to empower students as advocates to lead change initiatives and collaborate as co-creators of educational resources [23, 24]. The interest in ESD in the Middle East region is recent, in 2023 Jamal et al. conducted a study to determine the presence of ESD in the dental curricula of all dental schools in Saudi Arabia and the authors stated that ESD content is not formally embedded within the dental curricula in any dental institute in the country [25].

The United Arab Emirates (UAE) is a pioneer in creating and maintaining a sustainable environment and infrastructure in the Middle East region, in line with the pillars of the National Agenda of Vision 2021 [26]. Nonetheless, regardless of the country’s tremendous efforts towards environmental sustainability, there is a lack of contribution to these endeavors in the field of dentistry. Thus, this study aimed to explore the current awareness and drivers among dental students and educators regarding ESD in the UAE and identify barriers and enablers to embrace it in dental education in the country.

## Methods

To assess the awareness and drivers of the academic community of dental students and educators around the UAE towards ESD, a cross-sectional survey was carried out using online questionnaires that were circulated to all six undergraduate dental education institutes within the UAE. Ethical approval was obtained from the Research Ethics Committee at the University of Sharjah (REC-22-09-23-03-S). Invitations to participate were sent to all full-time dental educators (faculty) (*n* = 140), as well as all dental students at the undergraduate level of education (*n* = 1,900) in the UAE. The purpose of the study was explained to the study participants and written informed consent was obtained before commencing the survey.

The questionnaires used were from Joury et al. study [20]. The latter adapted the survey questionnaire for European dental schools that explored the understanding and teaching practice of sustainable healthcare. Joury et al. further improved the validity and reliability of the European questionnaire, created two questionnaires (one for students and the other for educators) and piloted the revised versions. Their questionnaires were used by other surveys such as Gershberg et al. 2022 and Jamal et al. 2023 [21, 25]. The present study made a few modifications to the questions, to ensure that they are appropriate for the UAE context. For example, some questions were removed for coherence with the UAE context and others were rephrased for clarity and comprehensiveness. The questionnaires began with a brief definition of the topic and introduction, where sustainability was defined as “the property of being environmentally sustainable; the degree to which a process or enterprise can be maintained or continued while avoiding the long-term depletion of natural resources”. The World Dental Federation 2017 Policy statement stated that: “Dentistry as a profession should integrate sustainable development goals into daily practice and support a shift to a green economy in the pursuit of healthy lives and well-being for all through all stages of life” [27].

Separate invitation emails were sent to the faculty and students of each institution. A reminder was sent by email twice between the period of November 2022 and March 2023. Data analysis included generation of summaries of the data by descriptive statistics.

## Results

In this study, the response rate was 8% among students and 37.8% among educators, with a total of 153 students and 53 educators participating. Only two participants provided free text (qualitative) responses. Hence, performing a thematic analysis on collected qualitative data was not possible.

Students’ ages ranged between 17 and 24 years, and their gender composition included a percentage of 32% male, and 68% female. As for educators, their age ranged from 28 to 64 years, and gender composition included 58.5% and 41.5% of male and female, respectively. Most educator participants were moderately (26.4%), somewhat (28.3%) or slightly familiar (28.3%) with the concept of ESD, while only 9.4% were extremely familiar and 7.5% were not familiar at all (Table 1). Among students, 26.8% of students were not familiar with the ESD concept and only 3.3% were extremely familiar (Table 1). Students largely agreed on the importance of ESD and their interest in learning it, as they viewed it relevant to their future practice (Tables 1 and 2). Educators also agreed on the importance of ESD and teaching it and expressed desire in introducing it into the dental curricula (Tables 1 and 2). One educator stated that “I would like to be actively involved in ESD”.

**Table 1:** Students’ and educators’ familiarity with ESD and their opinions about the importance and professional responsibility for ESD

Component	Overall	
	Students *N* (%)	Educators *N* (%)
**Familiarity with ESD**		
Not at all familiar	41 (26.8)	4 (7.5)
Slightly familiar	45 (29.4)	15 (28.3)
Somewhat familiar	37 (24.2)	15 (28.3)
Moderately familiar	25 (16.3)	14 (26.4)
Extremely familiar	5 (3.3)	5 (9.4)
**ESD is important**		
Strongly disagree	2 (1.3)	1 (1.9)
Disagree	2 (1.3)	Nil
Neutral	30 (19.6)	2 (3.8)
Agree	77 (50.3)	28 (52)
Strongly agree	42 (27.5)	22 (41.5)
**Profession’s responsibility for ESD**		
Strongly disagree	5 (3.3)	3 (5.7)
Disagree	1 (0.7)	Nil
Neutral	19 (12.4)	2 (3.8)
Agree	77 (50.3)	26 (49.1)
Strongly agree	51 (33.3)	22 (41.5)
**ESD teaching is important**		
Strongly disagree	5 (3.3)	1 (1.9)
Disagree	1 (0.7)	Nil
Neutral	30 (19.6)	2 (3.8)
Agree	69 (45.1)	22 (41.5)
Strongly agree	48 (31.4)	28 (52.8)
**Awareness of any ESD content being included in their dental curriculum**		
Yes	39 (25.5)	11 (20.8)
No	114 (74.5)	42 (79.2)

**Table 2:** Students’ and educators’ interest in ESD teaching and learning

Component	Overall	
	Students *N* (%)	Educators *N* (%)
**ESD relevance for future dental practice**		
Strongly disagree	3 (2)	-
Disagree	2 (1.3)	-
Neutral	35 (22.9)	-
Agree	68 (44.4)	-
Strongly agree	45 (29.4)	-
**Interested in learning ESD**		
Strongly disagree	3 (2)	-
Disagree	3 (2)	-
Neutral	39 (25.5)	-
Agree	75 (49)	-
Strongly agree	33 (21.6)	-
**Interested in introducing ESD into the dental curriculum**		
Strongly disagree	-	1 (1.9)
Disagree	-	Nil
Neutral	-	10 (18.9)
Agree	-	23 (43.4)
Strongly agree	-	19 (35.8)

Participants were asked about their awareness of any ESD content being included in the dental curriculum and it was found that 20.8% of educators and 25.5% of students thought that ESD content was present at their institution (Table 1). Those who reported the presence of ESD in the curriculum were also asked what ESD-related topics were covered (Table 3). Measuring and embedding sustainability into current dental practice and waste management got 8 out of 11 educator responses as examples of the covered topics, while sustainable equipment and supplies and energy saving were reported by 6 and 5 educators; respectively. Meanwhile, biodiversity and green space, travel and digital radiograph (other) got the least responses; 3, 2, and 1 educator; respectively. As for students, 23 out of 39 reported that energy conservation measures, such as turning off lights, monitors, and chairs when not in use were covered, while 22 students mentioned the presence of sustainable equipment and supplies topics. Meanwhile 18, 16, and 14 students indicated that waste management, measuring and embedding sustainability into current dental practice and travel were present in the curricula; respectively. The least frequently mentioned topic was biodiversity and green space, with 9 students selecting this answer. Educators and students who reported the presence of ESD-related topics in the curricula were further inquired about the format of content delivery, time devoted, assessment of student’s knowledge, and individuals responsible for the teaching ESD content. According to the 11 educators who reported the presence of ESD topics in the dental curricula, the most common forms of ESD-content delivery were lectures, seminars, projects and tutorials in a descending order. The most selected devoted time was 2–4 h (6 out of 8), followed by less than 2 h (3 out of 8), and more than 4 h (1 out of 8). One educator stated that there was no devoted individual for ESD teaching despite its presence in the curriculum. The mostly used formats of assessment according to educators were (in a descending order): multiple-choice questions, presentation, clinical evaluation, and written essay. One educator reported that students are taught but not assessed and another reported that students are neither taught nor assessed despite the presence of ESD-content in the curriculum (Table 3). Regarding the responsibility of teaching ESD content, all answers indicated that dental faculty of different courses were involved in the teaching.

**Table 3:** Students’ and educators’ responses on ESD topics in the dental curriculum, time dedicated, format of teaching and assessment

Component	Overall	
	Students (number of responses)	Educators (number of responses)
**ESD topics (select all that apply)**		
Travel	14	2
Equipment and supplies	22	6
Energy	23	5
Waste	18	8
Biodiversity and green space	9	3
Measuring and embedding sustainability into current dental practice	16	8
Other	Nil	1
**Time devoted for ESD in the curriculum**		
< 2 h	16	3
2–4 h	14	6
> 4 h	5	1
None	4	1
**Format of learning/teaching**		
Lecture	25	8
Seminar	8	5
Tutorial	16	3
Project	14	3
Other	3	2
**Format of assessment**		
Written essay	7	1
MCQs	24	8
Presentation	8	4
Clinical evaluation	13	4
Students are taught ESD but are not assessed	6	1
Students are not taught ESD nor assessed	3	1

According to the 39 students who reported the presence of ESD topics in the dental curricula, the most common forms of ESD-content delivery were (in a descending order): lectures, tutorials, projects, and seminars. The most selected devoted time was less than 2 h (16 out of 39), followed by 2–4 h (14 out of 39), and more than 4 h (5 out of 39). Four students responded to 0 time devoted for ESD teaching despite reported presence in the curricula. The mostly used formats of assessment according to students were (in a descending order): multiple-choice questions, clinical evaluation, presentation and written essay. Six students reported that they were taught but not assessed and three of them reported that they were neither taught nor assessed despite the presence of ESD-content in the curricula. Regarding the responsibility of teaching ESD content, most of the answers indicated that dental faculty of different courses were involved; however, 8 students reported that they did not know the answer for this question.

Table 4 summarizes the identified barriers and enablers by all educators for integrating ESD in the dental curricula. According to the educators, the most reported barrier is lack of knowledge about ESD, followed by insufficient curriculum space and resources for ESD educational materials. Other limitations included lack of time for preparing ESD content and not prioritizing ESD in the dental curriculum. The most reported factor that could enable the integration of ESD into the dental curricula was the provision of training courses for educators and teaching staff on ESD. Additionally, other important factors that were reported to enable the embedding of ESD in dental curricula included setting ESD-related learning outcomes and providing educators with time and resources to work on integrating ESD into dental curricula.

**Table 4:** Educators’ barriers and enablers to embedding ESD in the dental curricula

Barriers	Educators *N* (%)
Lack of knowledge	39 (73.6)
Lack of capacity/time	18 (34)
Lack of educational resources	20 (37.7)
Lack of priority	15 (28.3)
Lack of curriculum space	31 (58.5)
**Enablers**	
Learning outcomes	30 (56.6)
Training courses	45 (84.9)
Capacity/time	36 (67.9)
Presentations for students and staff	1 (1.9)

More than half of all students (54.9%) and the majority of educators (75.5%) emphasized the importance of incorporating ESD teaching in both classroom and clinical settings. One of the students stated: ‘I hope students and staff, people in general, will be more aware of ESD. They should know and be reminded of the importance of our environment. I hope our feedback will help you proceed with this goal’.

## Discussion

Following the publication of the consensus opinion arising from discussions at the ADEE sustainability workshop during the annual conference in Berlin in 2019, there has been a significant surge in interest in ESD. The consensus aimed to inform educators on the core principles outlined in the report on sustainable dental clinical practices and underscored the importance of integrating sustainability across the four educational domains and key competencies in general dentistry education [12]. This study was conducted to investigate, for the first time, the existing awareness and drivers among dental students and educators regarding ESD in the UAE. Additionally, the study sought to identify barriers and enablers influencing the adoption of ESD within dental education in the region.

Despite of the poor ESD awareness among students and educators in the UAE and the paucity of its official and clear inclusion in the current dental curricula shown in the current study, positive attitudes existed among most students and educators to adopt and integrate ESD into the curricula. These findings concur with numerous previous research in the literature on medical [28–30] and dental education [20–22, 25]. There is an evident awareness towards practical environmental protection within the young student generation, such as cycling and public transportation schemes, turning off energy-consuming equipment, and motion-sensing lights. However, significant barriers need to be addressed to integrate ESD in dental curricula including the lack of knowledge about ESD among students and educators, insufficient curriculum space and resources, lack of time for preparing the needed contents, and the tendency to not prioritize ESD learning outcomes in the dental curriculum. To overcome these barriers, this study recommends providing capacity building and training courses for educators and teaching staff on ESD “educate the educators”, integrating the subject in both classroom and clinical settings and focusing on setting ESD-related program objectives and learning outcomes. This list of recommendations does not necessarily require a complete overhaul of the educational program but rather incorporating ESD content into existing dental curricula. The barriers and enablers identified in this study share large similarities between sustainability-related barriers and enablers reported in previous studies. For instance, the results of our study are also in consonance with those obtained by Joury et al. in 2021 that explored ESD in dental schools in the US and UK [20]. Additionally, Gershberg et al. noted that there is limited room for additional curricular content. Nonetheless, their study findings indicated the feasibility of integrating ESD into existing courses [21]. A recent investigation conducted by Durnall et al. revealed that there is a notable interest and concern among undergraduate students in the UK on the topic of ESD. The abovementioned studies conducted in the UK served as a compelling catalyst for the incorporation of ESD into the academic curricula by the General Dental Council [20, 22]. In the USA, the situation mirrors that of the UK; Gershberg et al. highlighted the importance of assessing students’ interest and attitudes towards ESD to assist educators in devising effective strategies for its integration into the curriculum [21]. It is imperative to highlight that the primary emphasis should be on dental students, as they will play a pivotal role in fostering enduring sustainability-related attitudes and beliefs among future dental clinicians. The commencement of this endeavor should stem from their undergraduate dental education, serving as the foundation for instilling these values. Indeed, our study underscores the importance of students’ role as leaders and initiators for the promotion of environmental education. The enthusiasm among students for learning ESD, their acknowledgment of its relevance to their future practice, and the innate sense of responsibility towards the environment in younger generations suggest that engaging students in the planning and initiation of ESD activities and collaborations can pave the way for the successful implementation of ESD in both current curricula and future dental practice [20–22, 25]. Clinicians and students developing partnership in the learning process in this continuously developing field is of high importance [21]. The different institutes in the UAE that provide dental education can open the door to a shared collaboration between them to introduce ESD within their curricula, such agreement is a driver to accelerate the progress of ESD and the incorporation of the topic on a larger scope. Notwithstanding, funding, organizational factors and intellectual property challenges hinder such innovative measures that must take place in a wide spectrum, thus legislative authorities must address these challenges.

Similar to the findings of our study, Jamal et al. reported the desire of faculty to include ESD in their teaching, notwithstanding their limited foundational knowledge on the subject [25]. The efforts towards incorporating ESD in the dental curricula in the UAE can be facilitated by easing the way for educators to become competent in delivering ESD material through teaching techniques that students appreciate and benefit from such as Socratic seminars, reciprocal teaching and interdisciplinary learning. Moreover, laying the groundwork to achieve this goal require access to capacity building and training packages as well as creating a resource repository. Most available resources are medical based and do not fully encompass dentistry, or are outdated hence they need adaptation, modification and content update. Another innovative and practical method to educate dental faculty within the UAE on the importance of ESD is encouraging them to be part of associations and attend international conferences that are attempting to embed UNESCO’s SDGs into higher education [31, 32]. A further step is to increase their awareness on the country’s recent efforts for sustainable development, mainly in the healthcare sector such as Abu Dhabi’s healthcare sustainability goals announced in 2023, which aim to reduce carbon emissions with a focus on waste management [33].

Despite students’ and educators’ interest in ESD, having ESD legitimately embedded in the dental curricula in the UAE requires regulatory bodies such as the Ministry of Education (MOE), Commission for Academic Accreditation, Deans of UAE Colleges of Dentistry, Ministry of Health and Prevention and MOE National Qualification Centre to agree on incorporating sustainable dental practice into the learning outcomes, placing thereby an obligation on dental education providers to embed sustainability in their dental curricula. A structured Delphi approach could be ventured to identify a consensus view among dental students, educators, and other key allies about environmental sustainability learning outcomes in dental education and the possibility of making it part of the professional competency standards in the UAE [34]. Moreover, to advance environmental sustainability agenda in dental education and practice, more research is required on several aspects including the availability of materials, tools and equipment within the dental industry that comply with sustainability-related goals.

Despite the intriguing findings of the current study, there are several limitations. The low response rate might make it difficult to generalize the current findings to all students and educators at the various dental higher education institutes in the UAE. As such, it is possible that the present survey mainly included participants that were interested in and familiar with ESD. Nonetheless, the participants’ limited exposure to ESD, as demonstrated by their responses, actively works against such possibility. It is worth mentioning that previous surveys on environmental sustainability in dental education have also reported low response rates ranging between 37% and 5% [20–22, 25]. Lack of sufficient and in-depth qualitative data might be a further limitation. However, the present quantitative findings allowed the present study to achieve its aim in terms of exploring current ESD awareness and drivers among dental students and educators in the UAE and identify barriers and enablers to embrace it.

## Conclusion

There is no explicit ESD-related content in the undergraduate dental curricula at the dental higher education institutions in the UAE. Despite the lack of adequate awareness on the ESD topics among educators and more so within students, they both reported highly positive views towards incorporating ESD into the dental curricula. Overall, this study highlights the importance of promoting ESD in dental educational programs, as it can have significant benefits for both the environment and the future of dental profession in the UAE. The current findings provide valuable insights for educators, policymakers, and stakeholders on the potential barriers and enablers for integrating ESD into UAE dental curricula and can inform the development of strategies and initiatives to promote sustainability in dental education. This could be achieved by incorporating ESD into the UAE dental education requirements, legislative changes, and receiving institutional endorsement and support in the form of incentives, training, and academic resources.

## Data Availability

The authors confirm that the data supporting the findings of this study are available on request from the corresponding author.

## Abbreviations

ADEE: Association for Dental Education in Europe
CO_2_: Carbon dioxide
ESD: Environmentally sustainable dentistry
MOE: Ministry of education
SDG: Sustainable development goals
UAE: United Arab Emirates
UK: United Kingdom
UNESCO: United Nations Educational, Scientific and Cultural Organization
USA: United States of America
